# Transdiagnostic fears and avoidance behaviors in self-reported eating disorders

**DOI:** 10.1186/s40337-023-00745-8

**Published:** 2023-02-13

**Authors:** Hanna Melles, Anita Jansen

**Affiliations:** Department of Clinical Psychological Science, Faculty of Psychology and Neuroscience, Maastricht University, P.O. Box 616, 6200 MD Maastricht, The Netherlands

**Keywords:** Eating disorders, Anxiety, Avoidance behaviors, Transdiagnostic, Exposure therapy

## Abstract

**Background:**

Fears and avoidance behaviors are common symptoms of eating disorders. It was investigated whether different eating disorder diagnoses are equally characterized by similar fears and avoidance behaviors.

**Methods:**

Individuals with self-reported eating disorders (n = 250) and healthy controls (n = 95) completed online questionnaires assessing general fears, eating related fears, and avoidance behaviors.

**Results:**

All self-reported eating disorder diagnoses showed more eating related fears, general fears, and avoidance behaviors than healthy controls. Individuals with binge eating disorder showed less specific and general fears on some but by no means all scales, yet they showed less food avoidance behaviors than all other eating disorders and less eating restraint than anorexia nervosa and bulimia nervosa.

**Conclusions:**

Eating related fears, general fears, and food avoidance behaviors were found to be transdiagnostic symptoms in self-reported eating disorders. Individuals with binge eating disorder also exhibit more fears and avoidance behaviors than healthy controls, but to a lesser extent than the other eating disorders. Specialized interventions targeting fears and avoidance may be promising add-on interventions not only in the treatment of anorexia nervosa, but in the treatment of all eating disorders.

## Plain English summary

Individuals with anorexia nervosa suffer from a variety of fears such as the fear of gaining weight, losing control or being judged by others. An effective method to combat fear in the short term is using avoidance behaviors. Someone being afraid of never-ending weight gain may prevent the feared outcome by restricting their food intake. However, by doing this, the individual will never experience whether the expected and often unrealistic outcome actually occurs. In the long term, avoidance behaviors will therefore maintain those fears. In this study, we investigated whether all eating disorder diagnoses share the same specific fears, general fears and avoidance behaviors. Eating related fears, general fears, and food avoidance behaviors were found to be transdiagnostic symptoms in self-reported eating disorders. Individuals with binge eating disorder also exhibit more fears and avoidance behaviors than healthy controls, but to a lesser extent than the other eating disorders. Specialized interventions targeting fears and avoidance may be promising add-on interventions not only in the treatment of anorexia nervosa, but in the treatment of all eating disorders.

## Background

Anorexia nervosa (AN) is a difficult to treat eating disorder (ED): only about one third of the patients seeks help, dropout rates are substantial (~ 20–50%), recovery rates are modest (~ 30% within the first decade of the disorder) and relapse is high (~ 30%; [1–4]). Affected individuals restrict their caloric intake and show an unrelenting and obsessive drive towards thinness. This oftentimes results in alarmingly low body weights and life-threatening physical conditions. Despite the severity of anorexia nervosa, we do not know much about the mechanisms that maintain the disorder and impel patients in their endeavor to persistently lose weight, though fear was identified as an important and potentially perpetuating symptom already decades ago, and processes that create fears and avoidance may play a critical role (e.g. [5–13]). Studies show that patients with anorexia nervosa experience a large variety of fears such as fears of food and eating, fears of weight gain, fears of physical sensations like feeling satiated, fears related to personal and social consequences, as well as an increased intolerance of uncertainty, which is a fear-associated trait (see [11, 14–18]). There are indications that fear predates the onset of anorexia nervosa [19–21] has an impact on its course [22, 23] and predicts treatment outcomes [24, 25]. Patients with anorexia nervosa who are more anxious show a more severe course of the disorder and a worse prognosis than less anxious anorexia nervosa patients [8, 26, 27].

Fear related learning processes may play a role in the maintenance of anorexia nervosa [8, 10, 11, 28–33]. When eating is repeatedly associated with aversive outcomes, such as feeling bloated or weight gain, one learns to avoid the aversive outcomes and their predictors, like cues that predict the occurrence of eating [10]. Someone who is afraid of gaining weight or who feels guilty after eating high caloric foods easily can learn to avoid the food and eating, thereby preventing the expected aversive outcomes or the associated negative emotions. It has been argued that food avoidance and restrictive eating are behavioral responses to classically conditioned associations between food cues and aversive outcomes, making it one of the most destructive avoidance behaviors in anorexia nervosa [10, 30]. Avoidance temporarily reduces unpleasant fearful feelings, and this negative reinforcement will, in its turn, strengthen or maintain the avoidance behaviors [34–36]. The prevention of any kind of encounter with the fearful stimulus removes the possibility to experience that a feared aversive outcome might not become true. Therefore, in the longer term, avoidance perpetuates fears, and may even increase the fears [10, 37–40].

Fear is not only present in anorexia nervosa, it appears to be a common symptom amongst the other eating disorder diagnoses as well [27, 41–48]. For example, bulimia nervosa is associated with social anxiety (e.g. [49–51]) and individuals with binge eating disorder show fears as well (e.g. [52–54]). If fear is a transdiagnostic symptom of all eating disorders, it is to be expected that avoidance is a transdiagnostic mechanism in all eating disorders as well. Not only patients with anorexia nervosa but also individuals with other eating disorders apply a variety of avoidance behaviors. Next to food avoidance; emotional avoidance, body checking, body avoidance, wearing wide clothes; binge eating, vomiting and the excessive use of laxatives are just a few examples of common avoidance behaviors seen in individuals with eating disorders [11, 21, 30, 46, 55–60].

Though it is apparent from prior research that fear and avoidance do not only occur in anorexia nervosa but also in the other eating disorders, less is known about whether the different eating disorder diagnoses endorse the same (eating related) fears and whether they are of the same intensity. In the light of steadily low treatment outcomes of anorexia nervosa treatments [26, 61, 62] such knowledge could be relevant. Anxiety focused treatments such as exposure therapy aim at the disconfirmation of threat expectancies [63] and an essential pre-requisite to the choice of the right exposure target is a profound understanding of the fear-provoking cues and aversive outcomes a patient expects. So far, amongst the eating disorders, exposure therapy to feared foods, for instance, is most commonly applied in anorexia nervosa [12, 13, 28, 64–69]. If the other eating disorder diagnoses suffer from similar (eating related) fears as patients with anorexia nervosa, it is to be assumed that exposure therapy, which shows promising results in the treatment of anorectic fears, may be a valuable therapy for fear and avoidance in all eating disorders. The current study therefore aims to further investigate the transdiagnostic character of eating related fears, general fears and avoidance behaviors in eating disorders. Fear and avoidance are studied in both subtypes of anorexia nervosa (restrictive and binge purge), bulimia nervosa, binge eating disorder, and other specified feeding and eating disorders. It is hypothesized that all eating disorders show more eating related fear, general fear and avoidance behaviors than a healthy control group. In addition, from a transdiagnostic perspective, it is expected that the various eating disorder diagnoses do not differ from each other in the degree of fears and avoidance behaviors.

## Methods

### Participants

Female participants with self-reported eating disorders were recruited in several mental health/eating disorder centers in the Netherlands and Belgium (Emergis, Goes; Novarum, Amsterdam; Altrecht Rintveld, Utrecht; Alexianen, Tienen; Amarum, Nijmegen; PsyQ, Groningen; GGZ, Breburg; Co-eur, Roermond) and via the recruitment company Link2Trials. They were invited to participate in an online study on “emotions”. A minimum age of 16 years and an eating disorder diagnosis by a professional practitioner were required. In addition, a healthy control group of 16 years and older was recruited via flyers, advertisements on social media as well as the research participation system (SONA) of Maastricht University. Participants in the healthy control group were excluded when they were in psychological treatment currently or in the past three years, or when they reported to have a diagnosed mental disorder. This was assessed via a screening questionnaire at the beginning of the survey. After completion of the questionnaire, the participant received a 10€ online Amazon voucher.

### Measures

The following questionnaires were completed by the participants:

*Demographic data and DSM-5 criteria checklist.* Questions on current age, weight, length, received diagnosis, illness duration, treatment status, and treatment duration. All DSM-5 eating disorder criteria and symptoms (at the day of participation, in the past week, past month, past 3 months) were assessed with an unpublished diagnostic checklist by the second author that can be received upon request.

*Eating Disorder Examination Questionnaire (EDE-Q).* The EDE-Q 6.0 [70] assesses eating disorder psychopathology. The EDE-Q contains 28 items, each item is scored on a 0–6 scale, measuring the frequency or severity of eating disorder features over the past 28 days. Four subscales assessing eating restraint, eating concern, shape concern and weight concern are distinguished using 22 items, and their mean score reflects the global EDE-Q score. The remaining 6 items are open questions assessing binge-purge behaviors and excessive exercise. In the present study, the open questions were, amongst other instruments, used for diagnostic assessment of the participants. The four subscales are outcome measures, of which eating ‘restraint’ is considered an avoidance behavior (see “Food avoidance behaviors and eating restraint” section). Higher scores mean more severe eating disorder psychopathology. Internal consistencies in the present study are Cronbach’s α = 0.86 for eating restraint, α = 0.85 for eating concern, α = 0.89 for weight concern, α = 0.94 for shape concern and α = 0.97 for the global score.

*Fear of Food Measure (FOFM).* The FOFM [16] assesses fear of food. The self-report measure consists of 25 items that are rated from 1 to 7, and delivers three subscales: anxiety about eating (‘I feel tense when I am around food)’, feared concern (‘I worry that eating will make me dissatisfied with my body’) and food avoidance behaviors (‘There are certain foods I avoid because they make me anxious’). Item scores are summed to calculate the subscales. Higher scores mean more anxiety. Internal consistency of the subscales is Cronbach’s α = 0.96 for anxiety about eating, α = 0.92 for feared concern and α = 0.89 for food avoidance behaviors.

*Eating Disorder Fear Questionnaire (EFQ).* The EFQ [17] assesses eating disorder related fears. The measure consists of 20 items, rated from 1 to 7 and delivers five subscales: fear of weight gain (‘I am afraid of gaining weight’), fear of social consequences (‘I am afraid that I will be judged if I gain weight’), fear of personal consequences (‘I fear that I will lose control of my life if I gain weight’), physical sensations (‘I worry that I will not like how my body feels if I gain weight’) and fear of social eating (‘I am afraid of eating in public’). Item scores are averaged to calculate the subscales. Higher scores mean more anxiety. In the present study, internal consistency is Cronbach’s α = 0.94 for fear of weight gain, α = 0.95 for fear of social consequences, α = 0.92 for fear of personal consequences, α = 0.95 for fear of physical sensations and α = 0.96 for fear of social eating.

*Depression Anxiety Stress Scale (DASS).* The anxiety subscale of the short 21-item Dutch version of the DASS [71, 72] was used to assess general fear (‘I felt scared without any good reason’). Items are rated from 0 to 3. The subscale is calculated by summing the item scores and multiplying them by 2. Fear severity is categorized into normal (0–7), mild (8–9), moderate (10–14), severe (15–19) and extremely severe (20 +). Internal consistency in the present study is Cronbach’s α = 0.89.

*Intolerance of Uncertainty Scale (IUS).* The IUS [73] reflects how individuals react to ambiguous situations, implications of being uncertain as well as attempts to control the future (‘Uncertainty makes my life intolerable’). 27 items are rated from 1 to 5. Outcomes are summed to calculate a total score. Higher scores mean more intolerance of uncertainty. In the present study, internal consistency is Cronbach’s α = 0.96.

In addition, two self-made questionnaires on fear and body appreciation were added for potential development of new scales; they are not analyzed nor reported here.

### Procedure

The study was conducted online. After giving informed consent, the questionnaires (see “Measures” section) were completed in the following order: demographics; IUS; EFQ; FOFM; DASS; DSM-5 criteria checklist; body appreciation scale; fear scale and the EDE-Q, which took approximately one hour. Participants provided the diagnosis they received in their current or past treatment. The two authors independently inspected whether every self-reported diagnosis fitted within the overall clinical information that a participant provided in the survey. This information consisted of measures with established psychometric properties (EDE-Q), as well as an unpublished diagnostic checklist with which all DSM-5 eating disorder criteria and symptoms (at the day of participation, in the past week, past month, past 3 months) were assessed. Participants with anorexia nervosa were assigned to one of the two diagnostic subtypes (restrictive or binge purge). After this procedure was executed for every participant, the two authors compared their outcomes and discussed some cases. Self-reported diagnoses that did not fit a participant’s general clinical information were adjusted, e.g., when a patient reported binge eating and purge behaviors without a history of being underweight, bulimia nervosa seemed more appropriate than anorexia nervosa. Participants with anorexia nervosa who had a BMI higher than 18.5 at the moment of completing the questionnaire but who reported a history of being underweight (n = 23), retained the diagnosis anorexia nervosa. The study was approved by the Ethical Committee of the Faculty of Psychology and Neuroscience, Maastricht University, the Netherlands.

### Statistical analysis

All analyses were performed with JASP (version 0.16.3). Participants were excluded from the analysis when they completed less than one of the fear questionnaires. Participants in the healthy control group who showed eating disorder psychopathology on the EDE-Q (global score > 3) and/or the diagnostic scale (n = 16) were also excluded from the analyses. The sociodemographic data is described with means and standard deviations (SD), ranges or percentages. Bayesian analyses of variance (ANOVA) were used to evaluate whether the eating disorder diagnoses differed from the healthy control group and to test the equality hypotheses that there were no differences between the self-reported eating disorders. The Bayesian ANOVA yields a Bayes factor (BF) that quantifies the relative plausibility of two rival models [74, 75]. When testing the first hypothesis, the null model presumes no differences between the self-reported eating disorders and healthy controls, the alternative model states the opposite. When assessing the second hypothesis, the null model (no differences between the self-reported eating disorder diagnoses) was evaluated against the alternative model, assuming that the self-reported eating disorders differed from each other. BF01 is a Bayes factor that supports the null model and BF10 is a Bayes factor supporting the alternative model [74]. The higher a BF10, the more evidence for the alternative model. A BF10 = 10 means that the data are 10 times more likely under the alternative model than under the null model. When interpreting the Bayes factors the guidelines from Jeffreys [76] were applied (BF 1–3 = anecdotal; 3–10 = moderate; 10–30 = strong, 30–100 = very strong; > 100 = extreme evidence). In the results section, Bayes factors that yielded moderate evidence or more are reported. To account for multiple comparisons, the null control correction was applied. Outcomes of the post hoc tests are described in terms of corrected posterior odds. Single model R^2^ and 95% CI are reported as measure of effect size.

## Results

### Demographics, diagnosis and eating disorder psychopathology

The sample consisted of 345 participants of which 250 (72.5%) had a self-reported eating disorder diagnosis and 95 (27.5%) were healthy controls. Of the 250 participants with a self-reported eating disorder, n = 84 were diagnosed with anorexia nervosa restrictive subtype (AN-R, 33.6%), n = 57 with anorexia nervosa binge-purge subtype (AN-BP, 22.8%), n = 38 with bulimia nervosa (BN, 15.2%), n = 20 with binge eating disorder (BED, 8.0%) and n = 51 with other specified feeding and eating disorders (OSFED, 20.4%). The n = 23 patients with anorexia nervosa who had a BMI > 18.5 had a longer treatment duration than the remaining patients with anorexia nervosa (BF10 = 4.26, r^2^ = .04, 95% CI [.00, .11] but they did not differ on the other outcome measures. Of the participants with self-reported eating disorders, n = 70 (28.0%) were recruited directly via mental health institutions whereas the remaining n = 180 (72.0%) were recruited via Link2Trials. The Link2Trials sample entailed both treatment seeking individuals n = 95 (52.8%) and those who were not in treatment at the time of study participation n = 85 (47.2%). Thus, in the total eating disorder sample n = 165 (66%) individuals were in treatment. Participants recruited via clinics were older (BF10 > 100, r^2^ = .07, 95% CI [.02, .13]), had a longer illness duration (BF10 = 12.16, r^2^ = .04, 95% CI [.01, .09]) and more feared concerns (BF10 = 5.48, r^2^ = .03, 95% CI [.00, .08]) than participants recruited via Link2Trials. Further, treatment seeking individuals had higher IUS scores (BF10 = 18.92, r^2^ = .04, 95% CI [.01, .09]) and more eating anxiety than the non-treatment seeking sample (BF10 = 9.12, r^2^ = .04, 95% CI [.00, .09]).

The participants differed in BMI (BF10 > 100, r^2^ = .71, 95% CI [.68, .74]) in that individuals with binge eating disorder had a higher BMI than all the other self-reported eating disorder diagnoses and the healthy controls (all posterior odds > 100). Further, individuals with bulimia nervosa had a higher BMI than other specified feeding and eating disorders and both anorexia nervosa subtypes (all posterior odds > 100). The groups also differed in age (BF10 > 100, r^2^ = .09, 95% CI [.04, .14]). Participants with binge eating disorder were older than both anorexia nervosa subtypes and other specified feeding and eating disorders, and healthy controls were older than individuals with the anorexia nervosa binge purge subtype (all posterior odds > 100). Lastly, the self-reported eating disorder diagnoses differed in illness duration (BF10 > 100, r^2^ = .09, 95% CI [.03, .15]) in that participants with binge eating disorder had a longer illness duration than both anorexia nervosa subtypes and other specified feeding and eating disorders (posterior odds between 14.97 and > 100). Means and standard deviations and outcomes of the pairwise comparisons are displayed in Table 1.

**Table 1:** Demographic data

	AN-R (N = 84)	AN-BP (N = 57)	BN (N = 38)	BED (N = 20)	OSFED (N = 51)	HC (N = 95)
	M (SD) + range or %	M (SD) + range or %	M (SD) + range or %	M (SD) + range or %	M (SD) + range or %	M (SD) + range or %
Age	22.36 (5.59) 16–48	21.14 (4.54) 16–38	23.74 (7.98) 17–54	31.45 (11.16) 19–60	23.98 (6.48) 16–46	25.20 (8.10) 17–60
BMI (kg/m^2^)	16.69 (2.04) 11.0–21.6	16.53 (1.94) 11.0–19.8	21.69 (2.36) 18.6–28.3	36.49 (7.34) 25.2–49.8	21.46 (3.34) 13.8–33.3	22.54 (2.69) 18.5–30.48
Currently in treatment	72.6%	57.9%	60.5%	75%	64.7%	N.A
In treatment since (years)	3.82 (3.67) 1–26	4.45 (4.89) 1–25	3.43 (3.96) 1–23	4.40 (5.42) 1–21	4.22 (4.40) 1–20	N.A
Illness duration (years)	7.17 (5.54) 1–30	8.10 (5.63) 1–28	8.72 (6.83) 1–31	15.25 (9.65) 1–46	8.40 (5.97) 1–31	N.A

*AN-R*, anorexia nervosa restrictive subtype; *AN-BP*, anorexia nervosa binge-purge subtype; *BN*, bulimia nervosa; *BED*, binge eating disorder; *OSFED*, other specified feeding and eating disorder; *HC*, healthy controls

Participants with self-reported eating disorders were more concerned about eating (BF10 > 100, r^2^ = .58, 95% CI [.53, .62]), shape (BF10 > 100, r^2^ = .53, 95% CI [.47, .57]) and weight (BF10 > 100, r^2^ = .48, 95% CI [.42, .54]) than healthy controls. While the self-reported eating disorder diagnoses did not differ from each other in *shape concern* (BF01 = 3.53, r^2^ = .03, 95% CI [.01, .08]) they varied in *eating concern* (BF10 > 100, r^2^ = .10, 95% CI [.04, .18]). Individuals with other specified feeding and eating disorders (posterior odds between 33.56 and > 100) and the anorexia nervosa restrictive subtype (posterior odds between 3.12 and 33.21) were less eating concerned than participants with bulimia nervosa and the anorexia nervosa binge purge subtype. Means and standard deviations of the EDE-Q subscales are depicted in Table 2.

**Table 2:** Eating disorder psychopathology

EDE-Q	AN-R (N = 82)	AN-BP (N = 55)	BN (N = 35)	BED (N = 17)	OSFED (N = 40)	HC (N = 94)
Eating concern	2.97 (1.22)	3.60 (1.21)	3.94 (1.37)	3.27 (1.39)	2.60 (1.33)	0.38 (0.49)
Weight concern	4.06 (1.54)	4.57 (1.42)	4.73 (1.14)	4.22 (1.62)	3.97 (1.51)	1.32 (1.03)
Shape concern	4.58 (1.35)	4.99 (1.15)	4.83 (1.40)	4.57 (1.28)	4.30 (1.40)	1.65 (1.13)
Global score	3.76 (1.93)	4.29 (1.21)	4.37 (1.45)	3.66 (1.90)	3.57 (1.28)	1.07 (0.77)

*AN-R*, anorexia nervosa restrictive subtype; *AN-BP*, anorexia nervosa binge-purge subtype; *BN*, bulimia nervosa; *BED*, binge eating disorder; *OSFED*, other specified feeding and eating disorder; *HC*, healthy controls

### Fear: eating disorder specific fears

All self-reported eating disorder diagnoses scored higher than healthy controls (H1) and there were no differences between the eating disorder diagnoses (H2) on the EFQ subscales *fear of weight gain* (H1: BF10 > 100, r^2^ = .41, 95% CI [.35, .48]; H2: BF01 = 27.20, r^2^ = .02, 95% CI [.00, .05]), *fear of social consequences* (H1: BF10 > 100, r^2^ = .41, 95% CI [.35, .47]; H2: BF01 = 7.29, r^2^ = .03, 95% CI [.00, .07]), *fear of personal consequences* (H1: BF10 > 100, r^2^ = .55, 95% CI [.50, .60]; H2: BF01 = 18.68, r^2^ = .02, 95% CI [.00, .05]), *fear of physical sensations* (H1: BF10 > 100, r^2^ = .45, 95% CI [.39, .50]; H2: BF01 = 8.30, r^2^ = .03, 95% CI [.00, .07]), *fear of social eating* (H1: BF10 > 100, r^2^ = .39, 95% CI [.32, .45]; H2: BF01 = 13.06, r^2^ = .02, 95% CI [.00, .06]). Likewise, all self-reported eating disorder diagnoses scored higher than healthy controls on the FOFM subscales *anxiety about eating* (H1: BF10 > 100, r^2^ = .58, 95% CI [.53, .62]) and *feared concerns* (H1: BF10 > 100, r^2^ = .59, 95% CI [.54, .63]) but some self-reported eating disorders also differed from each other (H2: BF10 > 100 for *eating anxiety,* r^2^ = .08, 95% CI [.03, .15]; BF10 = 4.01 for *feared concerns,* r^2^ = .05, 95% CI [.01, .11]). Participants with binge eating disorder scored lower on both eating anxiety (posterior odds 19.90) and feared concerns (posterior odds 6.59) than individuals with the anorexia nervosa binge-purge subtype, and also lower on eating anxiety than the anorexia nervosa restrictive subtype (posterior odds 18.21) as well as bulimia nervosa (posterior odds 16.30). Individuals with other specified feeding and eating disorders were also less anxious about eating than participants with the anorexia nervosa binge-purge subtype (posterior odds 3.03) but on both scales they did not differ from the binge eating disorder diagnosis (posterior odds between 3.75 and 7.09). In addition, they also did not differ from the anorexia nervosa restrictive subtype (posterior odds 6.79) and bulimia nervosa (posterior odds 8.07) in feared concerns. Lastly, the anorexia nervosa subtypes did not differ from each other and they also did not differ from bulimia nervosa in anxiety about eating (posterior odds between 9.37 and 14.15) and feared concerns (posterior odds between 3.18 and 14.98). Means and standard deviations of the EFQ and FOFM subscales and the outcomes of the pairwise comparisons are shown in Table 3.

**Table 3:** Specific eating fears measured with the EFQ and FOFM

EFQ	AN-R (N = 84)	AN-BP (N = 57)	BN (N = 38)	BED (N = 20)	OSFED (N = 49)	HC (N = 94)
Fear of weight gain	6.08 (1.29)	6.24 (1.29)	6.46 (0.87)	6.20 (1.09)	6.11 (1.37)	3.57 (1.68)
Fear of social consequences	5.06 (1.57)	5.41 (1.51)	5.47 (1.44)	5.92 (1.22)	5.36 (1.46)	2.60 (1.34)
Fear of personal consequences	5.53 (1.34)	5.64 (1.47)	5.55 (1.29)	5.14 (1.56)	5.22 (1.43)	2.07 (1.23)
Fear of physical sensations	6.26 (1.25)	6.50 (1.03)	6.35 (0.96)	6.25 (1.03)	5.97 (1.35)	3.48 (1.81)
Fear of social eating	4.51 (1.98)	5.10 (2.19)	4.43 (2.10)	5.05 (1.55)	4.57 (1.92)	1.51 (0.97)

FOFM	AN-R (N = 84)	AN-BP (N = 57)	BN (N = 37)	BED (N = 19)	OSFED (N = 46)	HC (N = 89)
Anxiety about eating	38.71 (11.02)^b,c,*d*^	41.05 (13.10)^a,c,*d*,*e*^	40.97 (11.71)^a,b,*d*^	28.10 (13.69)^*a−c*,e^	33.67 (11.86)^*b*,d^	10.59 (4.74)
Feared concerns	42.26 (11.67)^b,c,e^	46.21 (12.38)^a,c,*d*^	42.08 (10.99)^a,b,e^	35.84 (10.44)^*b*,e^	39.26 (12.14)^a,c,d,^	14.10 (5.69)

Different lettered superscripts were used to indicate which eating disorder diagnoses differed from each other and which were equal at a statistical level of > 3 for the corrected posterior odds. The groups are coded as follows: AN-R = a, AN-BP = b, BN = c, BED = d, OSFED = e. Letters written italic represent group differences. Non-italic letters represent equality

*AN-R*, anorexia nervosa restrictive subtype; *AN-BP*, anorexia nervosa binge-purge subtype; *BN*, bulimia nervosa; *BED*, binge eating disorder; *OSFED*, other specified feeding and eating disorder; *HC*, healthy controls

To sum up, all self-reported eating disorders showed more eating disorder specific fears than healthy controls. The diagnostic categories did not differ in specific eating fears on five out of seven subscales, but participants with binge eating disorder showed less fears about eating than those with anorexia nervosa and bulimia nervosa as well as less feared concerns than individuals with the anorexia nervosa binge purge subtype. Anorexia nervosa and bulimia nervosa did not differ from each other and neither did binge eating disorder differ from other specified feeding and eating disorders. The latter also did not differ in feared concerns from the anorexia nervosa restrictive subtype and bulimia nervosa.

### Fear: general fears

The self-reported eating disorder categories differed from each other on the DASS anxiety subscale (H2: BF10 = 17.85, r^2^ = .06, 95% CI [.02, .13]), but they all scored higher than the healthy control group (H1: BF10 > 100, r^2^ = .29, 95% CI [.22, .36]). Participants with binge eating disorder showed less fear than those with the anorexia nervosa binge purge subtype (posterior odds 25.34) but they did not differ from the other specified feeding and eating disorders (posterior odds 3.24). The latter also did not differ from the anorexia nervosa restrictive subtype (posterior odds 14.44). Lastly, individuals with bulimia nervosa did not differ from both anorexia nervosa subtypes (posterior odds between 5.96 and 9.75) and other specified feeding and eating disorders (posterior odds 6.16). In addition, the self-reported eating disorder diagnoses did not differ from each other in intolerance of uncertainty (H2: BF01 = 5.24, r^2^ = .03, 95% CI [.01, .07]), while they all scored higher on intolerance of uncertainty than healthy controls (H1: BF10 > 100, r^2^ = .31, 95% CI [.24, .37]). Means and standard deviations of the DASS and IUS and outcomes of the pairwise comparisons are displayed in Table 4.

**Table 4:** General fears measured with the DASS and IUS

DASS	AN-R (N = 84)	AN-BP (N = 56)	BN (N = 36)	BED (N = 18)	OSFED (N = 46)	HC (N = 87)
Anxiety	19.31 (11.64)^c*,*e^	24.82 (11.62)^c,*d*^	21.50 (10.27)^a,b,e^	12.89 (11.73)^*b*,e^	18.30 (10.95)^a,c,d^	6.28 (6.61)

IUS	AN-R (N = 84)	AN-BP (N = 57)	BN (N = 38)	BED (N = 20)	OSFED (N = 51)	HC (N = 95)
	93.24 (18.63)	96.21 (21.39)	88.68 (19.85)	89.00 (23.88)	87.57 (18.91)	62.57 (17.84)

Different lettered superscripts were used to indicate which eating disorder diagnoses differed from each other and which were equal at a statistical level of > 3 for the corrected posterior odds. The groups are coded as follows: AN-R = a, AN-BP = b, BN = c, BED = d, OSFED = e. Letters written italic represent group differences. Non-italic letters represent equality

*AN-R*, Anorexia nervosa restrictive subtype; *AN-BP*, anorexia nervosa binge-purge subtype; *BN*, bulimia nervosa; *BED*, binge eating disorder; *OSFED*, other specified feeding and eating disorder; *HC*, healthy controls

### Food avoidance behaviors and eating restraint

Healthy controls reported fewer food avoidance behaviors compared to all self-reported eating disorder diagnoses (H1: BF10 > 100, r^2^ = .55, 95% CI [.49, .60]). Additionally, participants with binge eating disorder reported less food avoidance behaviors than the other eating disorder diagnoses (H2: BF10 > 100, r^2^ = .14, 95% CI [.07, .21]; posterior odds for both anorexia nervosa subtypes > 100; bulimia nervosa = 38.16; other specified feeding and eating disorders = 42.72). The anorexia nervosa subtypes did not differ from each other in food avoidance behaviors (posterior odds 16.10). Bulimia nervosa did not differ from the anorexia nervosa binge-purge subtype (posterior odds 3.44) and from the other specified feeding and eating disorders (posterior odds 13.58). Healthy controls reported less eating restraint compared to all eating disorder diagnoses (H1: BF10 > 100, r^2^ = .43, 95% CI [.37, .49]) and individuals with binge eating disorder reported less eating restraint than the anorexia nervosa binge purge subtype (H2: BF10 = 4.455, r^2^ = .05, 95% CI [.01, .11]; posterior odds 5.76) and bulimia nervosa (posterior odds 16.77). Participants with anorexia nervosa (posterior odds between 3.60 and 15.27) and bulimia nervosa (posterior odds 3.61) did not differ from the other specified feeding and eating disorders. Further, individuals with the anorexia nervosa binge purge subtype did not differ from bulimia nervosa (posterior odds 13.83). Means and standard deviations of the FAB subscale and eating restraint subscale and outcomes of the pairwise comparisons are provided in Table 5.

**Table 5:** Avoidance behaviors measured with the FAB and the EDE-Q

FOFM	AN-R (N = 84)	AN-BP (N = 57)	BN (N = 37)	BED (N = 19)	OSFED (N = 46)	HC (N = 89)
FAB	31.14 (7.56)^b,*d*^	30.65 (9.15)^a,c,*d*^	27.35 (7.87)^b,*d*,e^	18.53 (7.78)^*a*,*b*,*c*,*e*^	27.22 (8.07)^c,*d*^	10.84 (5.99)

EDE-Q	AN-R (N = 82)	AN-BP (N = 55)	BN (N = 35)	BED (N = 17)	OSFED (N = 40)	HC (N = 83)
Eating restraint	3.42 (1.46)^e^	3.98 (1.63)^c,*d*,e^	3.96 (1.22)^b,*d*,e^	2.58 (1.35)^*b*,*c*^	3.39 (1.55)^a−c^	0.94 (1.00)

Different lettered superscripts were used to indicate which eating disorder diagnoses differed from each other and which were equal at a statistical level of > 3 for the corrected posterior odds. The groups are coded as follows: AN-R = a, AN-BP = b, BN = c, BED = d, OSFED = e. Letters written italic represent group differences. Non-italic letters represent equality

*AN-R*, anorexia nervosa restrictive subtype; *AN-BP*, anorexia nervosa binge-purge subtype; *BN*, bulimia nervosa; *BED*, binge eating disorder; *OSFED*, other specified feeding and eating disorder; *HC*, healthy controls

## Discussion

It was investigated whether individuals with various self-reported eating disorder diagnoses share similar eating related fears, general fears, and avoidance behaviors. The hypotheses were largely supported: All self-reported eating disorders show more eating related fears, general fears, and avoidance behaviors than healthy controls. The eating disorder diagnoses did not differ in eating related fears on five out of seven scales, though individuals with binge eating disorder were less anxious about eating than individuals with anorexia nervosa and bulimia nervosa, and they showed less feared concerns than anorexia nervosa. While the self-reported eating disorders did not differ from each other in intolerance of uncertainty, participants with binge eating disorder were less anxious than participants with anorexia nervosa in general anxiety measured with the DASS. Individuals with binge eating disorder also showed less food avoidance behaviors than all other self-reported eating disorders and less eating restraint than anorexia nervosa and bulimia nervosa.

Considering that all self-reported eating disorder diagnoses robustly show general and eating related fears, it is intriguing that fears, anxiety, and avoidance are often not specifically targeted in eating disorder treatments. Fear may be a maintenance mechanism [10] and therefore a critical treatment target across eating disorders. The gold standard treatment for anxiety-related disorders is exposure therapy based on inhibitory learning, which aims at the disconfirmation of threat expectancies [63]. During the exposure intervention, core fears and threat expectancies of patients are formulated into hypotheses, and it is tested whether expected aversive outcomes actually occur. Knowing a patient’s feared stimuli and expected aversive outcomes very precisely is integral to the success of an exposure intervention. The application of exposure therapy in eating disorders is not new but the use of exposure and exposure protocols is scarce (see [55]). So far, the targeted treatment of eating related fears, such as the fear of food, (over)eating, and weight gain, by means of exposure therapy takes place only sparsely; it is not usually an explicit goal of current eating disorder therapies even though several pilot studies already revealed promising results of exposure therapy on fear reduction in anorexia nervosa [12, 13, 28, 66–69] and clinical exposure protocols are available (e.g. [64]). The main fears targeted in exposure therapy are fears of food and weight gain since they are considered core fears in anorexia nervosa [21, 26, 31, 32]. Especially in the context of weight restoration, which is a crucial element of recovery, anorexia nervosa patients could profit from confronting their irrational fears about weight gain and for example learn that eating a meal will not cause infinite weight gain [10]. The present study confirms that those fears are not only endorsed by individuals with anorexia nervosa; they are symptoms of all eating disorders. This study also shows that fears in self-reported eating disorders are heterogeneous in that patients do not only fear food, eating, and weight gain; they may also be very fearful of social eating situations and the perceived social consequences of eating (being rejected, judged, criticized), personal consequences (becoming lazy, losing control) and physical sensations (feel emotionally or physically uncomfortable) because of food, eating and weight gain. The outcomes of eating disorder treatments can be improved when core fears are more narrowly targeted by (add-on) exposure interventions and this seems to be true for all eating disorders.

Next to eating related fears, avoidance behaviors are transdiagnostic eating disorder characteristics though individuals with binge eating disorders show less avoidance behaviors and attitudes than the other self-reported eating disorders. The avoidance behaviors assessed in this study mainly involve food avoidance and eating restraint, making it less surprising that individuals with binge eating disorder, who engage in binge eating episodes without inappropriate compensatory behaviors, do not show more avoidance.

Our study has some limitations. A first limitation is the small range of avoidance behaviors that have been assessed. Individuals with binge eating disorder may apply avoidance behaviors that were not investigated here, and others suggest that the binge eating behavior itself is an avoidance strategy to regulate emotions and negative self-evaluations [46, 56, 57]. Important other avoidance behaviors in eating disorders are body checking and body avoidance [60, 77–79]. A second limitation is the small size of the binge eating disorder sample, which requires careful interpretation of those findings, as well as their replication. Third, the sample of participants with self-reported eating disorders entailed a high proportion of treatment seeking individuals which may contribute to a high prevalence of fears. However, the likelihood of this seems small given that fear and avoidance in the subsample not in treatment is just as strong. Further, the sample consisted of females only and was exclusively recruited in the Netherlands and Belgium. Finally, clinical eating disorder diagnoses, given by the practitioner, were reported by the patients themselves and confirmed or corrected by the authors using self-reported symptoms. It is vital to replicate the current findings using a clinical diagnostic interview.

## Conclusion

General fears, eating related fears and avoidance behaviors are omnipresent in self-reported eating disorders, though the latter is less evident in the binge eating disorder. The new insights from this study should stimulate future research on eating disorder maintaining mechanisms and exposure-based treatments, targeting fear and avoidance in all eating disorders.

## Data Availability

The datasets used and/or analyzed during the current study are available from the corresponding author on reasonable request.
